# Repositioning antipsychotic chlorpromazine for treating colorectal cancer by inhibiting sirtuin 1

**DOI:** 10.18632/oncotarget.4768

**Published:** 2015-09-05

**Authors:** Wen-Ying Lee, Wai-Theng Lee, Chia-Hsiung Cheng, Ku-Chung Chen, Chih-Ming Chou, Chu-Hung Chung, Min-Siou Sun, Hung-Wei Cheng, Meng-Ni Ho, Cheng-Wei Lin

**Affiliations:** ^1^ Department of Pathology, Chi Mei Medical Center, Tainan, Taiwan; ^2^ Department of Pathology, School of Medicine, College of Medicine, Taipei Medical University, Taipei, Taiwan; ^3^ Department of Biochemistry and Molecular Cell Biology, School of Medicine, College of Medicine, Taipei Medical University, Taipei, Taiwan; ^4^ Graduate Institute of Medical Sciences, School of Medicine, College of Medicine, Taipei Medical University, Taipei, Taiwan; ^5^ Institute of Cellular and Organismic Biology, Academia Sinica, Taipei, Taiwan

**Keywords:** chlorpromazine, drug reposition, p53, apoptosis, SIRT1

## Abstract

Investigating existing drugs for repositioning can enable overcoming bottlenecks in the drug development process. Here, we investigated the effect and molecular mechanism of the antipsychotic drug chlorpromazine (CPZ) and identified its potential for treating colorectal cancer (CRC). Human CRC cell lines harboring different p53 statuses were used to investigate the inhibitory mechanism of CPZ. CPZ effectively inhibited tumor growth and induced apoptosis in CRC cells in a p53-dependent manner. Activation of c-jun N-terminal kinase (JNK) was crucial for CPZ-induced p53 expression and the subsequent induction of tumor apoptosis. Induction of p53 acetylation at lysine382 was involved in CPZ-mediated tumor apoptosis, and this induction was attenuated by sirtuin 1 (SIRT1), a class III histone deacetylase. By contrast, knocking down SIRT1 sensitized tumor cells to CPZ treatment. Moreover, CPZ induced the degradation of SIRT1 protein participating downstream of JNK, and JNK suppression abrogated CPZ-mediated SIRT1 downregulation. Clinical analysis revealed a significant association between high SIRT1 expression and poor outcome in CRC patients. These data suggest that SIRT1 is an attractive therapeutic target for CRC and that CPZ is a potential repositioned drug for treating CRC.

## INTRODUCTION

Colorectal cancer (CRC) is the most common cancer and the second cause of cancer-related death worldwide. Genetic mutation is the major cause of colorectal oncogenesis, and *TP53* tumor suppressor gene mutation occurs in approximately 40%–60% of patients with colon cancer [1]. The protein p53 (encoded by the *TP53* gene) plays a crucial role in preventing cancer development and progression by inducing growth arrest, senescence, or apoptosis or by impeding tumor migration, invasion, or angiogenesis. Approximately 75% of *TP53* gene mutations are point mutations, which lead to amino acid substitutions and result in the inhibition of normal p53 function and loss of suppressor function [2].

Several cellular stresses, such as oxidative stress, hypoxia, DNA damage, and chemotherapeutics, can activate p53. Once activated, p53 can execute its cellular functions through a transcription-dependent or -independent mechanism. In p53-dependent apoptosis, p53 transactivates proapoptotic genes, including *BAX*, *PUMA*, *NOXA*, and *P21*^Waf1/Cip1^. The p53 protein level can be negatively modulated by MDM2 and MDMX, two E3 ubiquitin–protein ligases that mediate p53 protein degradation through ubiquitin-dependent proteolysis [3]. Other posttranslational modifications of p53, such as acetylation, sumoylation, and phosphorylation, also regulate p53 activity. Acetylation of p53 by p300/CREB binding protein prevents MDM2-mediated p53 degradation and increases p53 activity [4, 5]. By contrast, deacetylation of p53 by deacetylases such as histone deacetylase (HDAC) and sirtuin 1 (SIRT1) destabilizes it and facilitates MDM2-mediated protein degradation [6, 7]. Although wild-type p53 is expressed in approximately 50% of patients with cancer, it remains dysfunctional in these tumor cells because of abnormally elevated levels of p53 suppressors such as MDM2, MDMX, SIRT1, and HDACs [8–10]. Therefore, several studies have emphasized the need to discover small molecules that can inhibit p53 suppressors for potential application in therapies for p53-harboring cancers [11–13].

Developing new drugs for cancer treatment is a time- and cost-intensive process. Unexpected toxicity at any stage may cause failure during development. Therefore, examining existing drugs for drug repositioning is an effective strategy for accelerating drug development and reducing investment risk [14]. Numerous studies have scanned existing drugs for new indications in cancer therapy. For instance, the antiinflammatory drug licofelone suppressed pancreatic cancer progression [15]. Cholesterol biosynthesis inhibitors, statins, suppressed tumor growth and metastases in several types of cancer cells [16–18]. The type 2 diabetes drug metformin exhibited potent antitumor activity against breast and brain cancer stem cells [19–22]. These findings indicate that repositioning drugs is a promising approach for cancer treatment. With a recent advance in genomics, the Connectivity Map database reveals potential connections between small molecules and diseases. Among these small molecules, antipsychotic drugs exhibited tumor suppression activity [23, 24]; however, their detailed inhibitory activity and mechanism remain unclear. Chlorpromazine (CPZ) is an FDA-approved phenothiazine derivative used to treat schizophrenia and other psychiatric disorders. The pharmacological mechanism of CPZ in schizophrenia treatment is mediated through the blockage of dopamine receptors. However, CPZ also exhibited antiproliferative activity against brain tumors, probably through the suppression of AKT/mTOR activation or the upregulation of p21^Waf1/Cip1^ expression [25, 26]. However, the effect of CPZ on CRC and its inhibitory mechanism have not been reported.

This study investigated the molecular mechanism of CPZ in CRC treatment. We observed that CPZ-induced tumor apoptosis depended on p53 protein expression. CPZ-induced p53 upregulation and acetylation were mediated through c-Jun N-terminal kinase (JNK)-dependent SIRT1 inhibition in CRC cells.

## RESULTS

### Identification of CPZ as a potent anti-colon-cancer drug

**Figure 1:**
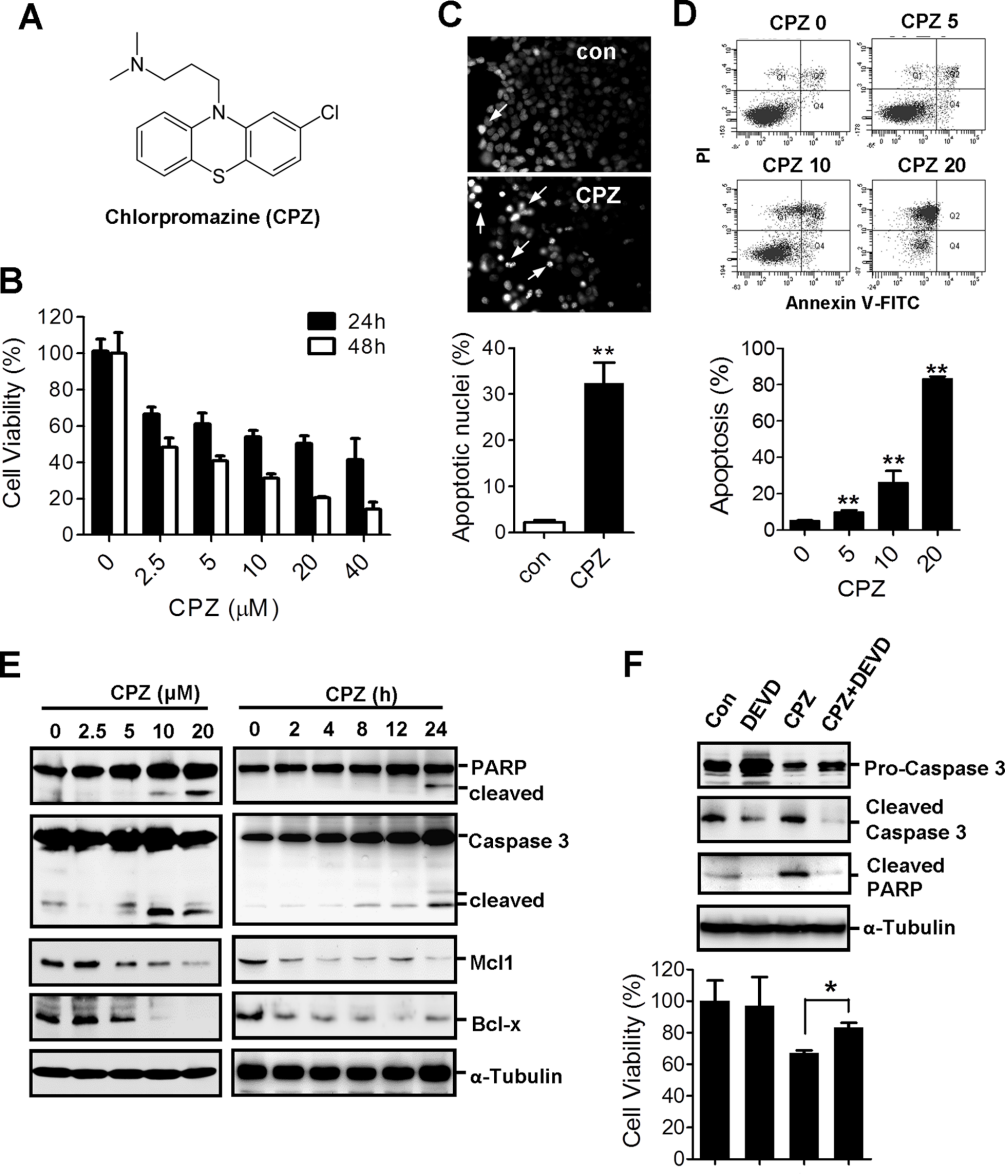
Induction of apoptosis in human CRC cells by CPZ **A.** Chemical structure of CPZ. **B.** Reduction in cell viability of HCT116 cells in the presence of CPZ (0–40 μM; 24 and 48 h). Cell viability was assessed using an MTT assay. **C.** CPZ-induced chromatin condensation and apoptosis in HCT116 cells as determined through DAPI staining. **D.** The induction of tumor apoptosis by CPZ was determined using flow cytometry after annexin V-PI staining. **E.** HCT116 cells were treated with different CPZ concentrations (0–20 μM) for 24 h or with 10 μM CPZ for different time periods, and apoptotic proteins were analyzed using Western blotting. **F.** HCT116 cells were treated with CPZ (10 μM) in the presence of a caspase 3 inhibitor (z-DEVD-FMK, 20 μM) for 24 h, and caspase 3 and PARP protein expression was analyzed using Western blotting. Cell viability was assessed using an MTT assay. *P < 0.05 and **P < 0.01 indicate significant differences.

CPZ (Figure 1A) is a phenothiazine derivative used to treat psychotic disorders. To determine whether CPZ has antitumor activity against CRC, HCT116 cells were treated with 2.5–40 μM CPZ for 24 and 48 h. Results of an MTT assay revealed that cell viability substantially decreased after CPZ treatment. CPZ IC_50_ values at 24 and 48 h were 11.6 and 3.7 μM, respectively (Figure 1B). By contrast, CPZ exhibited no obvious cytotoxic effect on normal lung fibroblast WI38 cells up to a concentration of 20 μM (Supplementary Figure S1). To determine whether CPZ causes tumor apoptosis, HCT116 cells were stained with 4, 6-diamidino-2-phenylindole (DAPI). CPZ treatment induced a significant increase in condensed nuclei, an indicator of cellular apoptosis (Figure 1C). Results of flow cytometry obtained using annexin V-propidium iodide (PI)-stained cells further revealed that treatment with 5–20 μM CPZ increased tumor apoptosis from 10% to 83% compared with that of untreated cells (Figure 1D). Correspondingly, apoptotic protein markers, as characterized by an increase in PARP and caspase 3 cleavage and a decrease in the levels of the antiapoptotic proteins Mcl-1 and Bcl-x, were detected in a concentration- and time-dependent manner after CPZ treatment (Figure 1E). However, pretreatment with a caspase 3 inhibitor, z-DEVD-FMK, suppressed CPZ-induced PARP and caspase 3 cleavage and suppressed cell death (Figure 1F).

### CPZ induces p53-dependent apoptosis in CRC

**Figure 2:**
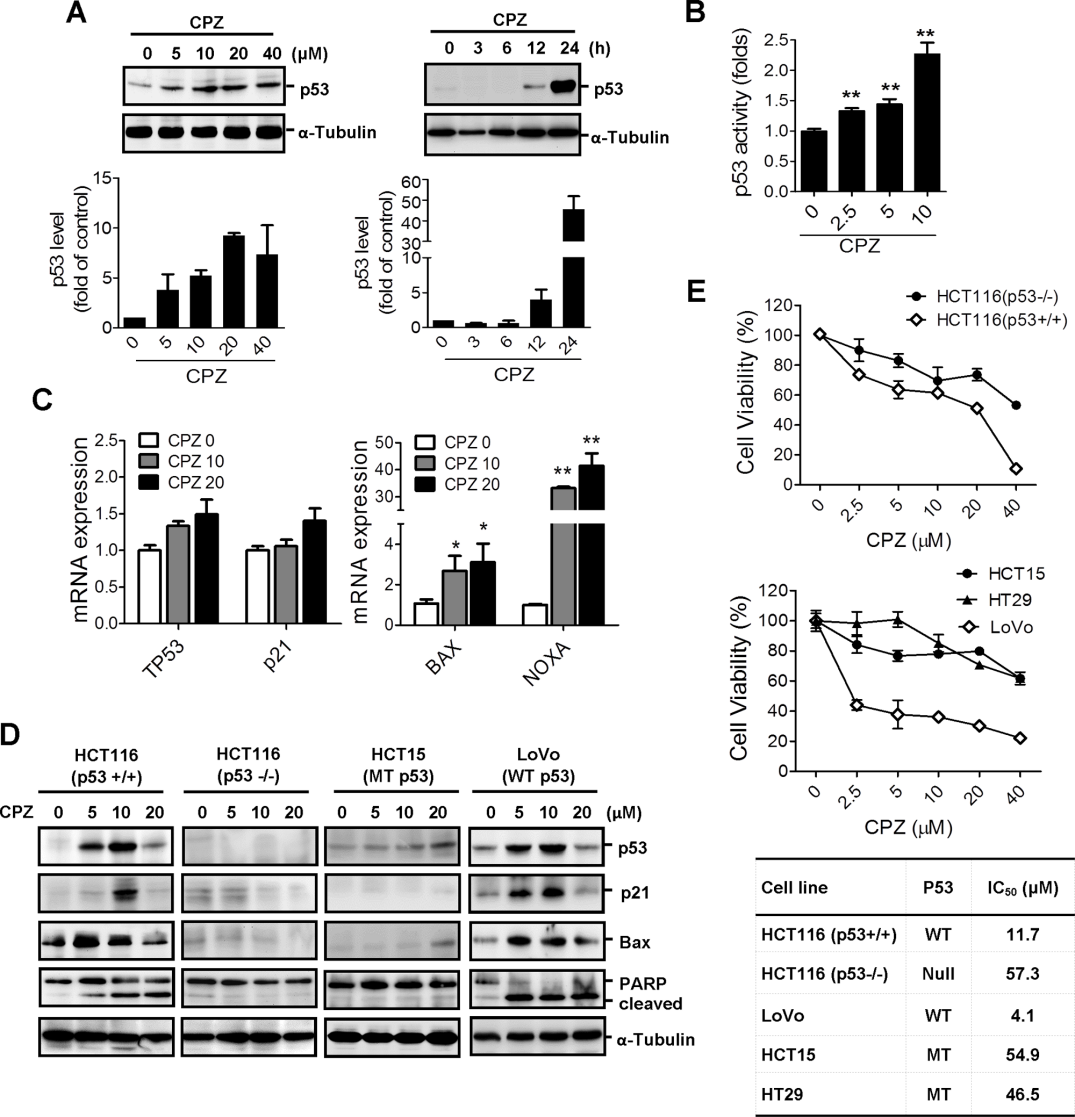
Induction of p53-dependent apoptosis in human CRC cells by CPZ **A.** Concentration- and time-dependent induction of p53 protein expression by CPZ in HCT116 colon cancer cells. Cells were treated with CPZ (0–40 μM) for 24 h or with 10 μM CPZ for different time periods. The p53 protein level was analyzed using Western blotting and was quantified using ImageJ software. Data are expressed as the mean ± SE and represented as fold changes relative to the control, *n* = 3. **B.** HCT116 cells were transfected with the PG13-luc plasmid and then treated with CPZ for 24 h. The transcriptional activity of p53 was measured using a luciferase assay. **P < 0.01 indicates a significant difference. **C.** Induction of p53-dependent gene (*TP53*, *p21*, *BAX*, and *NOXA*) expression by CPZ (10 μM) treatment for 24 h as determined through real-time PCR analysis. **D** and **E.** CPZ-mediated tumor suppression depended on the p53 protein level. HCT116 (wild-type p53), HT29 (mutated p53), HCT15 (mutated p53), LoVo (wild-type p53), and HCT116 p53 − /− cells were incubated with CPZ for 24 h, the expression of p53 and p53-regulated proteins was assessed using Western blotting (D) and cell viability was assessed using an MTT assay (E).

To determine whether CPZ induces tumor apoptosis through a p53 mechanism, we treated HCT116 cells with CPZ and analyzed them using Western blotting. The results revealed that CPZ induced p53 protein expression in a dose- and time-dependent manner (Figure 2A). In addition, CPZ treatment dose-dependently increased p53 transcriptional activity (Figure 2B) and induced the expression of p53 downstream target genes, including *TP53*, *p21*, *BAX*, and *NOXA* (Figure 2C). CPZ slightly increased p53 and p21 mRNA levels and significantly induced *BAX* and *NOXA* expression. To verify that p53 is crucial for CPZ-mediated cell death, we analyzed the responses of p53 and its downstream targets p21^Waf1/Cip1^, BAX, and PARP to CPZ in different CRC cell lines. As expected, protein levels of p53, p21waf1/Cip1, Bax, and cleaved PARP increased in HCT116 and LoVo cells, which contain wild-type p53. However, this effect was not observed in p53-null HCT116 (p53 − / − ) and HCT15 (MT p53) cells (Figure 2D). Similarly, results of a cell viability assay showed that HCT116 (p53+/+) and LoVo (WT p53) cells were more susceptible to CPZ than HCT116 (p53 − / − ), HCT15 (MT p53), and HT29 (MT p53) cells were (Figure 2E). These data indicate that p53 participates in CPZ-mediated cell death.

### JNK activation is crucial in CPZ-mediated p53 expression and tumor apoptosis

**Figure 3:**
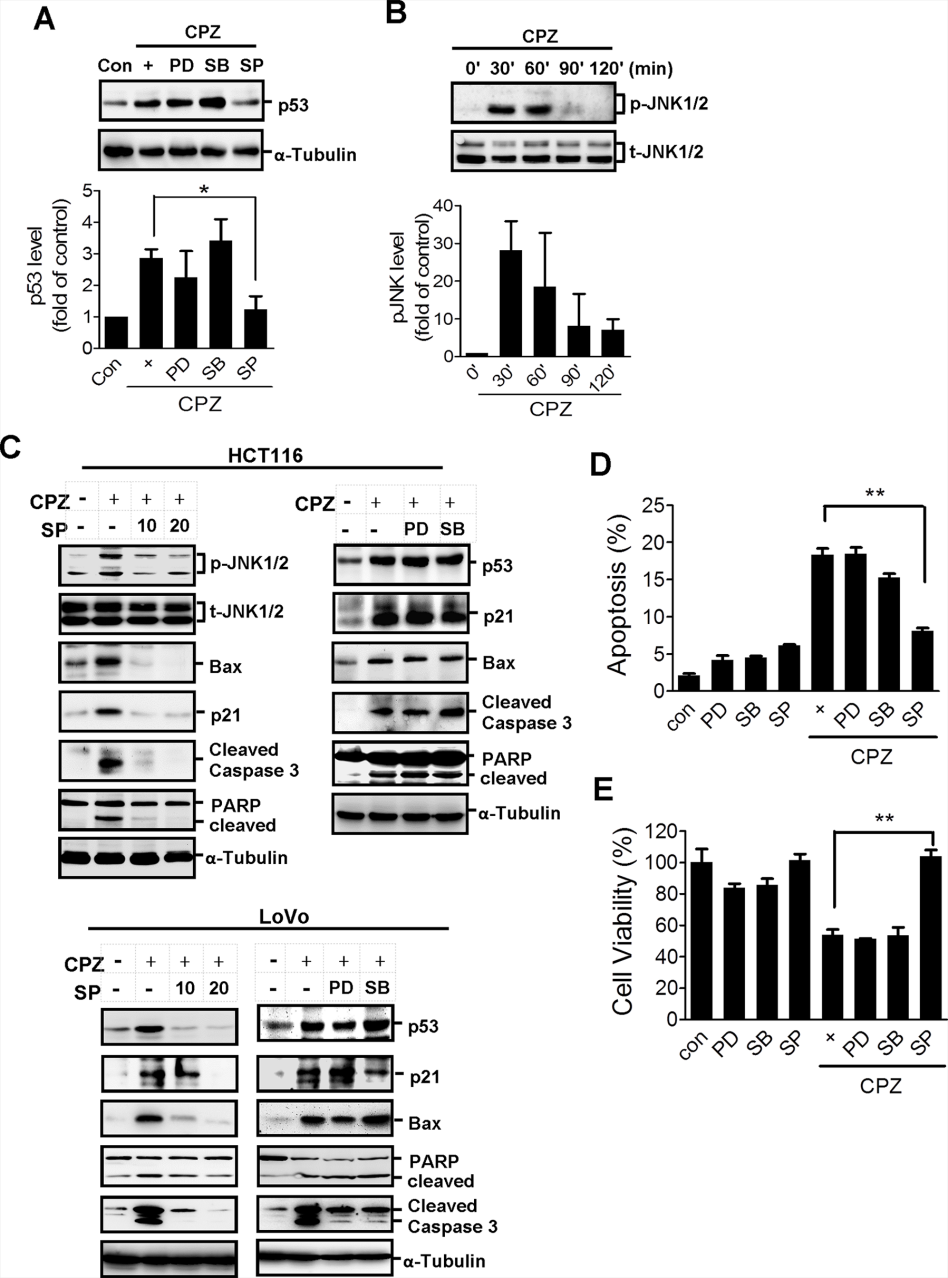
JNK activation is involved in CPZ-mediated p53 expression **A.** HCT116 cells were treated with CPZ (10 μM) in the presence of an ERK inhibitor (PD98059, PD: 20 μM), a JNK inhibitor (SP600125, SP: 20 μM), and a p38 inhibitor (SB203580, SB: 20 μM). The expression of p53 protein was analyzed using Western blotting and quantified using ImageJ software. *P < 0.05 indicates a significant difference. **B.** Western blot analysis of JNK protein phosphorylation after CPZ (10 μM) treatment. **C–E.** Suppression of JNK, but not ERK or p38, diminished CPZ-induced tumor apoptosis. HCT116 and LoVo cells were treated with CPZ (10 μM) in the presence of PD98059 (20 μM), SB203580 (20 μM), or SP600125 (10 and 20 μM) for 24 h, protein expression was analyzed using Western blotting (C) and tumor apoptosis (E) and cell viability (D) were measured using annexin V-PI and MTT assays, respectively.

Several lines of evidence have identified the importance of the mitogen-activated protein kinase (MAPK) family in p53 regulation [27]. To investigate the signaling underlying CPZ-induced p53 expression, we treated cells with inhibitors of the MAPK family. Pretreatment with the JNK inhibitor SP600125 abrogated CPZ-induced p53 protein expression (Figure 3A). Treatment with PD98059 and SB203580, inhibitors of ERK and p38, respectively, did not suppress p53 protein expression (Figure 3A). In addition, JNK phosphorylation was detected in CPZ-treated cells (Figure 3B), and SP600125 pretreatment suppressed CPZ-induced JNK phosphorylation, which was accompanied by a reduction in apoptotic protein levels elicited by CPZ (Figure 3C). By contrast, neither PD98059 nor SB203580 treatment suppressed CPZ-induced p53 and apoptotic protein expression in both HCT116 and LoVo cells (Figure 3C). Consistently, treatment with SP600125, but not SB203580 or PD98059, significantly blocked CPZ-induced cytotoxicity in HCT116 cells (Figures 3D and 3E). Overall, these data suggest that JNK activation is crucial for CPZ-mediated tumor apoptosis.

### CPZ induces p53 acetylation, which is repressed by SIRT1

**Figure 4:**
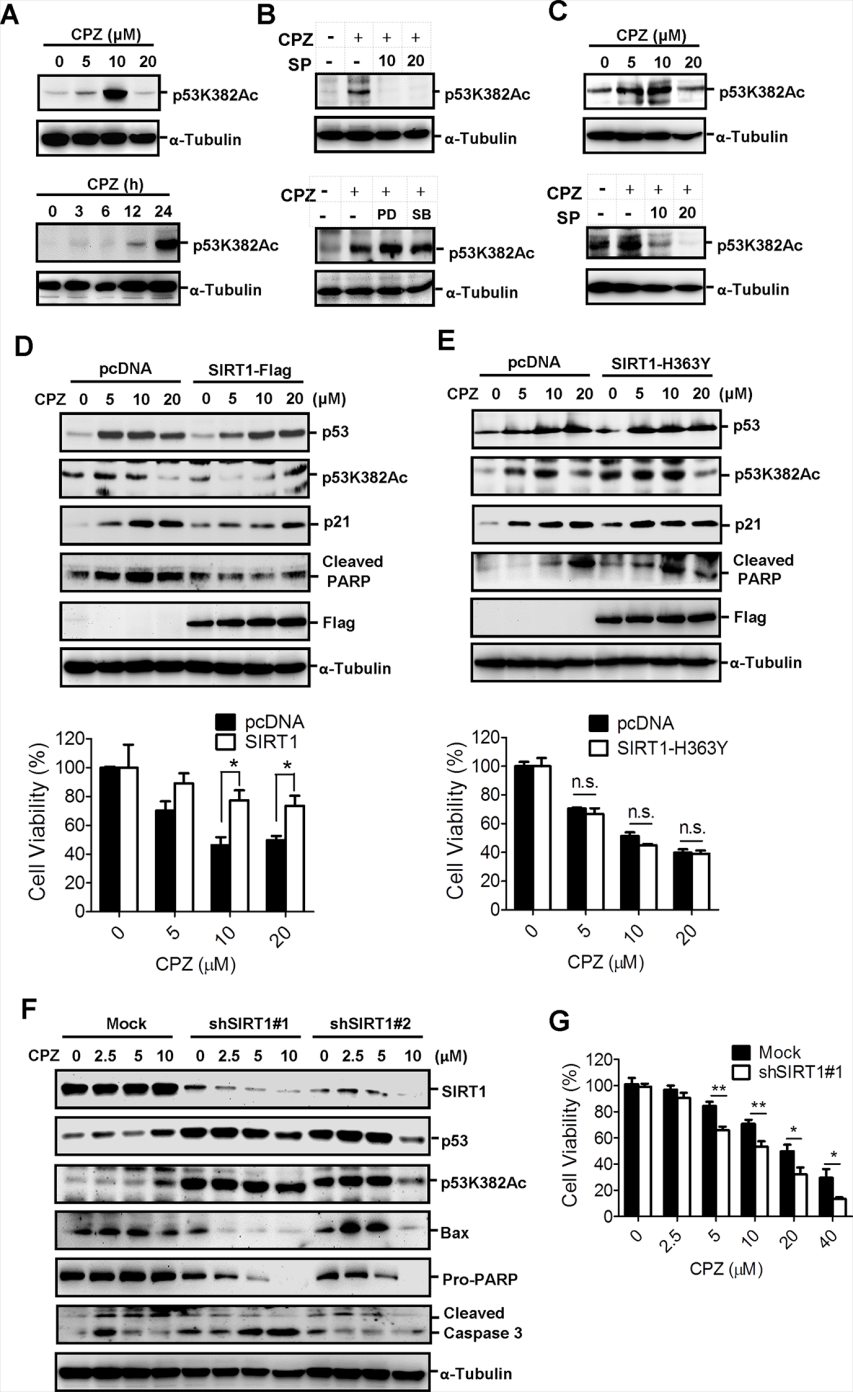
Induction of p53 acetylation at Lys382 by CPZ, which was attenuated by SIRT1 expression **A.** Concentration- and time-dependent induction of p53 acetylation in CPZ-treated HCT116 cells. Acetylated p53 was analyzed through Western blotting by using specific antiacetylated p53 Lys382 (p53K382Ac) antibodies. **B.** HCT116 cells were treated with CPZ (10 μM) in the presence of SP600125, PD98059, or SB203580 (20 μM) for 24 h. Acetylated p53 was analyzed using Western blotting. **C.** CPZ induced p53 Lys382 acetylation, which was blocked by SP600125. LoVo cells were treated with CPZ (0–20 μM, left panel) or CPZ and SP600125 for 24 h, and p53 Lys382 acetylation was detected using Western blotting. **D** and **E.** SIRT1 overexpression reduced CPZ-elicited cell death. HCT116 cells were transfected with the wild-type SIRT1-Flag (D) or mutant SIRT1-H363Y-Flag (E) plasmids for 24 h and treated with CPZ (0–20 μM). Protein expression was analyzed using Western blotting, and cell viability was assessed using an MTT assay. *P < 0.05 indicates a significant difference. **F** and **G.** Suppression and SIRT1 sensitized cells to CPZ-induced tumor apoptosis. HCT116/mock and SIRT1-knockdown cells were treated with CPZ (0–10 μM) for 24 h, protein expression was analyzed using Western blotting (F) and cell viability was measured using an MTT assay (G).

Previous studies have reported that the acetylation of p53 increases its transcriptional activity; therefore, we investigated whether CPZ affects p53 acetylation. HCT116 and LoVo cells were treated with CPZ and subjected to Western blot analysis using a specific antiacetylated p53 lysine382 (Lys382) antibody. Results showed that CPZ induced p53 Lys382 acetylation in a dose- and time-dependent manner (Figures 4A and 4C upper panel). However, p53 Lys382 acetylation was inhibited by the addition of SP600125, but not PD98059 or SB203580 (Figures 4B and 4C lower panel). Because Lys382 is specifically deacetylated by SIRT1, we examined whether SIRT1 overexpression diminishes the effect of CPZ. SIRT1 overexpression affected the p53 protein level to a lesser extent than that observed in mock cells, whereas it substantially reduced CPZ-induced p53 Lys382 acetylation and affected downstream targets of p53, reducing p21^Waf1/Cip1^ expression and inducing PARP cleavage (Figure 4D upper panel). Moreover, CPZ-elicited growth inhibition was restored in SIRT1-overexpressing cells (Figure 4D lower panel). By contrast, overexpression of SIRT1-H363Y plasmid, a mutated SIRT1 without deacetylase activity, did not prevent CPZ-induced p53 Lys382 acetylation and protein expression of p53, p21^Waf1/Cip1^, and PARP (Figure 4E upper panel). Similarly, the overexpression of mutated SIRT1 failed to protect the cells from CPZ-mediated cell death (Figure 4E lower panel).

To further delineate the crucial role of SIRT1 in suppressing CRC apoptosis, SIRT1 was knocked down in HCT116 cells and the cells were treated with CPZ. The results indicated that CPZ treatment induced p53 acetylation and protein expression in mock knockdown cells. However, CPZ treatment of SIRT1-knockdown cells failed to induce further p53 protein expression and acetylation. SIRT1 silencing robustly increased the basal level and acetylation of p53 protein without CPZ treatment (Figure 4F), and these data were consistent with previous findings that SIRT1 negatively regulates p53 acetylation and protein stability. Nevertheless, knocking down SIRT1 markedly enhanced CPZ-induced apoptotic protein expression (Figure 4F) and enhanced susceptibility to CPZ-elicited cell death (Figure 4G).

### CPZ induces SIRT1 protein degradation

**Figure 5:**
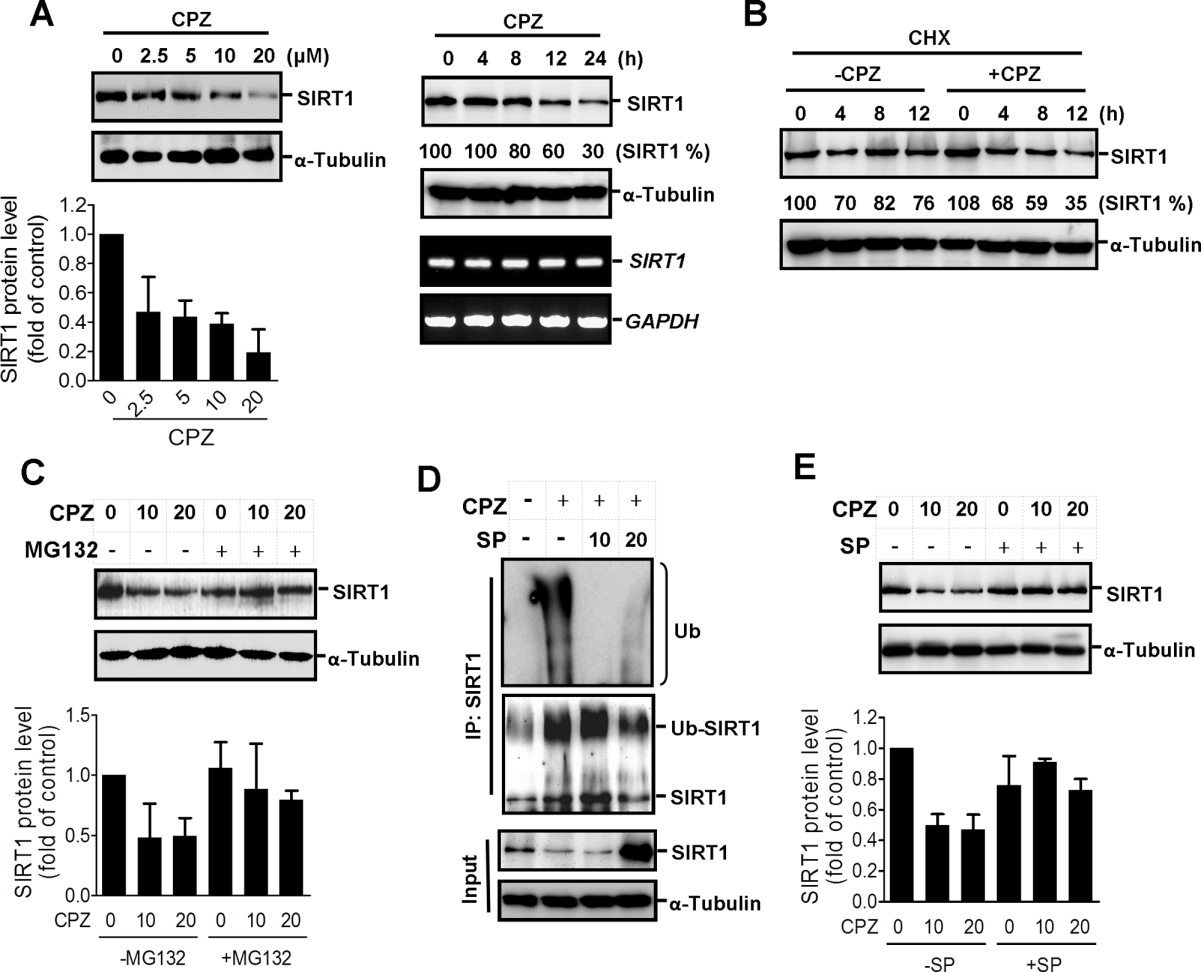
Induction of SIRT1 protein degradation by CPZ **A.** HCT116 cells were treated with CPZ in a dose- and time-dependent manner, and SIRT1 protein and mRNA levels were analyzed using Western blotting and RT-PCR assays, respectively. SIRT1 protein expression was quantified using ImageJ software. **B.** HCT116 cells were treated with cycloheximide (CHX, 50 μg/mL) in the presence or absence of CPZ (10 μM) for 0–12 h, and the SIRT1 protein level was analyzed using Western blotting. **C.** HCT116 cells were treated with CPZ (0, 10, and 20 μM) in the presence or absence of MG132 (25 μM), and SIRT1 protein expression was analyzed using Western blotting. **D.** HCT116 cells were pretreated with SP600125 (10 and 20 μM) and then treated with CPZ for 24 h. Protein lysates were immunoprecipitated with an antiSIRT1 antibody and blotted with antiubiquitin (upper panel) and anti-SIRT1 (lower panel) antibodies. **E.** HCT116 cells were pretreated with SP600125 (20 μM) and then incubated with CPZ (10 and 20 μM) for 24 h, and the SIRT1 protein level was assessed using Western blotting and quantified using ImageJ software.

To clarify the regulatory mechanism of CPZ in p53 acetylation, we analyzed the effects of CPZ on SIRT1. The results of Western blotting showed that CPZ treatment reduced the SIRT1 protein level in a dose-dependent manner (Figure 5A left panel). SIRT1 protein expression decreased substantially 12 h after CPZ treatment, whereas the SIRT1 mRNA level was unaffected by CPZ treatment (Figure 5A right panel), suggesting that CPZ attenuates SIRT1 protein stability. To test this hypothesis, HCT116 cells were pretreated with cycloheximide, a protein synthesis inhibitor, and then treated with CPZ. As expected, CPZ promoted SIRT1 protein degradation in the presence of cycloheximide (Figure 5B), and the CPZ-elicited decrease in SIRT1 expression was recovered in the presence of the proteasome inhibitor MG132 (Figure 5C). Moreover, results of coimmunoprecipitation analysis revealed that CPZ induced SIRT1 ubiquitination; however, SP600125 pretreatment suppressed SIRT1 ubiquitination (Figure 5D and Supplementary Figure S2A) and diminished CPZ-mediated SIRT1 suppression (Figure 5E). These results indicate that CPZ induction of p53 acetylation and tumor apoptosis might be mediated through SIRT1 protein level suppression.

### CPZ suppresses the growth of human xenograft colon cancer

**Figure 6:**
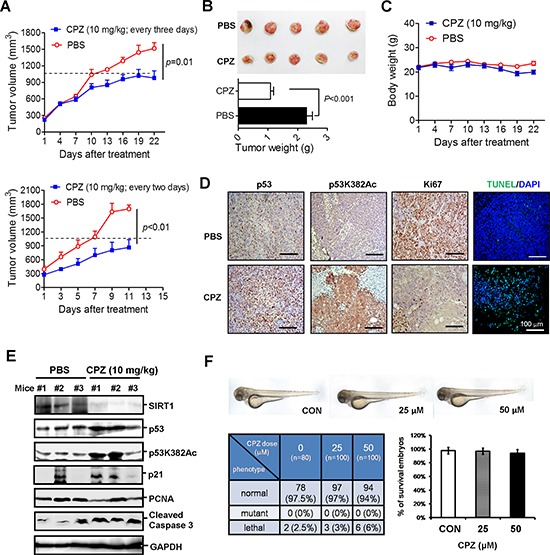
Suppression of colon cancer growth *in vivo* by CPZ **A.** HCT116 cells (2 × 10^6^) were subcutaneously injected into the dorsal flank of NOD/SCID mice. When the tumor volume reached 300 mm^3^, the mice were administered CPZ (10 mg/kg) every 3 days (upper panel) or 2 days (lower panel) and the tumor volume, weight of xenograft tumors **B.** and body weight of mice **C.** receiving CPZ every 3 days were measured. The results are expressed as the mean ± SE, *n* = 6. **D.** Immunohistochemical analysis of p53, p53K382Ac, and Ki67 protein expression in CPZ-treated (every 2 days) tumor sections. Apoptotic tumor cells were analyzed using a TUNEL assay. Scale bar = 100 μm. **E.** Western blotting of protein levels in CPZ- (10 mg/kg, every 2 days) and PBS-treated tumor extracts. **F.** Assessment of CPZ toxicity in zebrafish embryonic development.

To further assess the *in vivo* tumor suppression activity of CPZ, we injected HCT116 (p53+/+) cells into nonobese diabetic/severe combined immunodeficiency (NOD/SCID) mice. Once the average tumor volume reached 300 mm^3^, the mice were administered 10 mg/kg CPZ intravenously every 3 days for 3 weeks. The tumor volume grew substantially; however, the growth was slower in the CPZ-treated group than in the PBS-treated group (*P* = 0.01) (Figure 6A upper panel). CPZ significantly reduced the tumor volume at the end of the experiment by nearly 50% (*P* < 0.01; Figure 6B). The mice receiving CPZ were healthy and exhibited no apparent changes in body weight throughout the experimental period (Figure 6C). We conducted another experiment in which the mice were administered 10 mg/kg CPZ every 2 days for 2 weeks; compared with tumor growth in the control group, that in the CPZ-treated group was significantly suppressed (*P* < 0.01; Figure 6A lower panel). We further analyzed p53 protein levels in xenograft tumor sections, and results of an immunohistochemistry assay revealed that CPZ treatment increased p53 protein expression and p53K382 acetylation (Figure 6D). Conversely, the proliferative cell marker Ki67 decreased in CPZ-treated tumors (Figure 6D). In addition, CPZ treatment induced tumor apoptosis, as observed in terminal-deoxynucleotidyl-transferase-mediated dUTP nick-end labeling (TUNEL) assays (Figure 6D). An analysis of the protein levels in tumor extracts yielded similar results; CPZ reduced SIRT1 and PCNA protein expression, whereas it increased p53 and p21 expression, p53K382 acetylation, and caspase 3 cleavage (Figure 6E).

CPZ has been extensively used in clinics for treating schizophrenia, indicating that it is safer than conventional chemotherapeutic agents; nevertheless, we examined whether the dose used in cancer treatment had side effects on embryonic development. We used a zebrafish model to monitor the effects of CPZ on embryonic development. The results showed that treating zebrafish embryos with 25 or 50 μM CPZ for 48 h yielded a survival rate similar to that observed during the embryonic development of wild-type zebrafish (*n* = 80 in control group and *n* = 100 in CPZ-treated group), and no significant phenotypic change between vehicle- and CPZ-treated zebrafish embryos was observed throughout the experiment (Figure 6F). Overall, these data demonstrate that CPZ effectively suppresses tumor growth and induces apoptosis.

### Clinical relevance of SIRT1 expression in CRC

**Figure 7:**
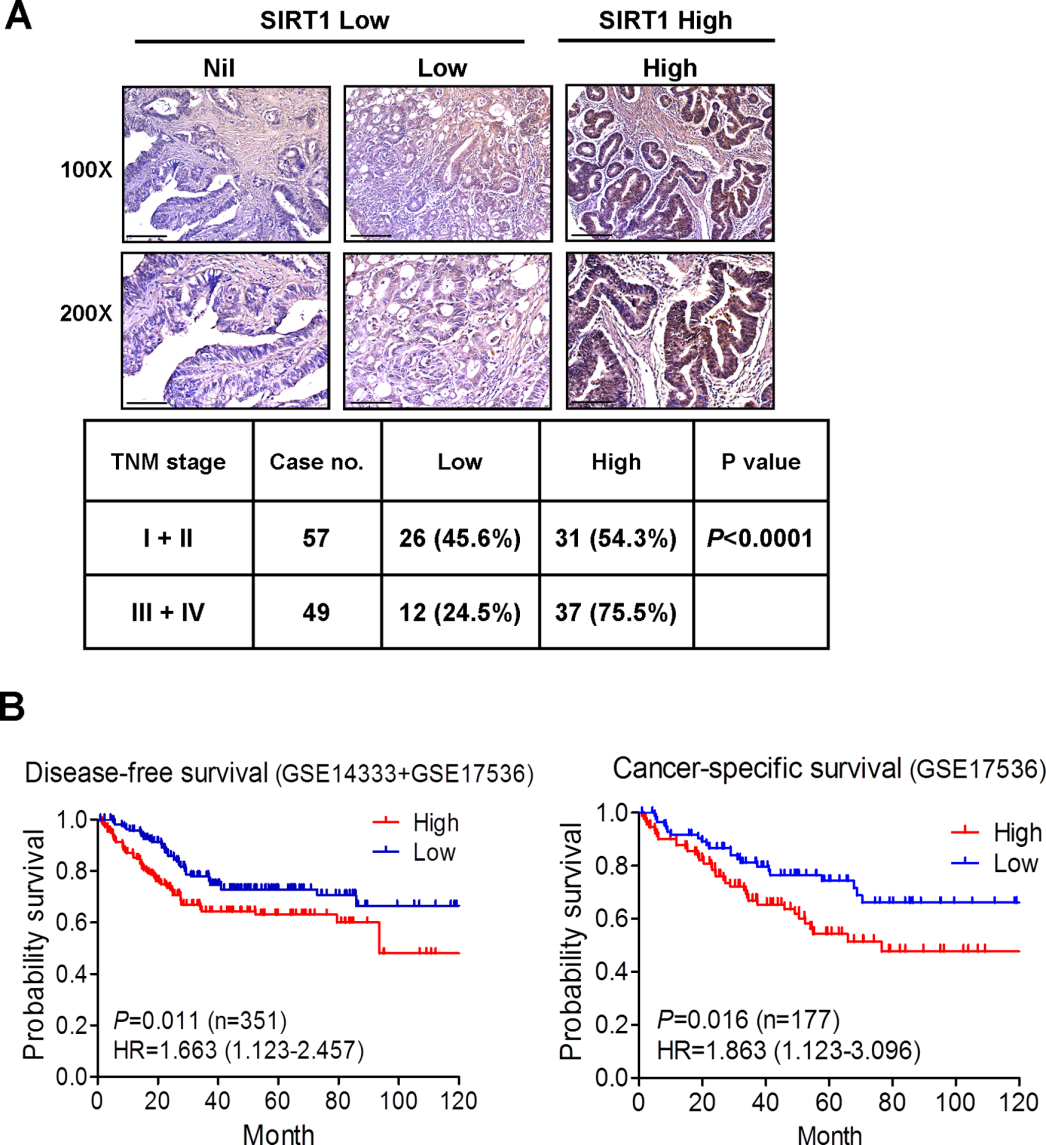
Clinical relevance of SIRT1 expression in CRC **A.** Immunohistochemical staining of SIRT1 in the CRC tissue microarray. Representative IHC data showing CRC with nil, low, and high SIRT1 expression. Scale bar = 100 μm in the upper panel and 50 μm in the lower panel. The *P* value was determined using the Fisher's exact test. **B.** Kaplan–Meier curves representing the association of the SIRT1 mRNA level with the probability of disease-free survival (pooled GSE14333 and GSE17536 CRC data sets) and cancer-specific survival (GSE17536 CRC data sets). *P* values were analyzed using the log-rank statistical test.

To validate the clinicopathological role of SIRT1 in CRC progression, we examined the SIRT1 protein level in a CRC tissue microarray by using immunohistochemistry. SIRT1 was highly expressed in approximately 65% (69 of 106) of CRC specimens, as demonstrated by strong immunoreactivity (Figure 7A). Moreover, SIRT1 protein expression was significantly elevated in advanced tumor stages (75%, 37 of 49 cases) compared with that in stage I + II (54%, 31 of 57 cases) (Figure 7A). We also analyzed the prognostic value of the SIRT1 mRNA level on the basis of two independent cohort studies and observed a significant correlation between high SIRT1 expression and decreased disease-free survival (pooled GSE14333 and GSE17536 CRC data sets, *P* = 0.011; HR = 1.663, 95% CI of ratio = 1.123–2.457; *n* = 351). Notably, patients with a high SIRT1 level exhibited poor cancer-specific survival (GSE17536 CRC data sets, *P* = 0.016; HR = 1.863, 95% CI of ratio = 1.12 − 3.09; *n* = 177) (Figure 7B). These data suggest that high SIRT1 expression contributes to CRC progression.

## DISCUSSION

**Figure 8:**
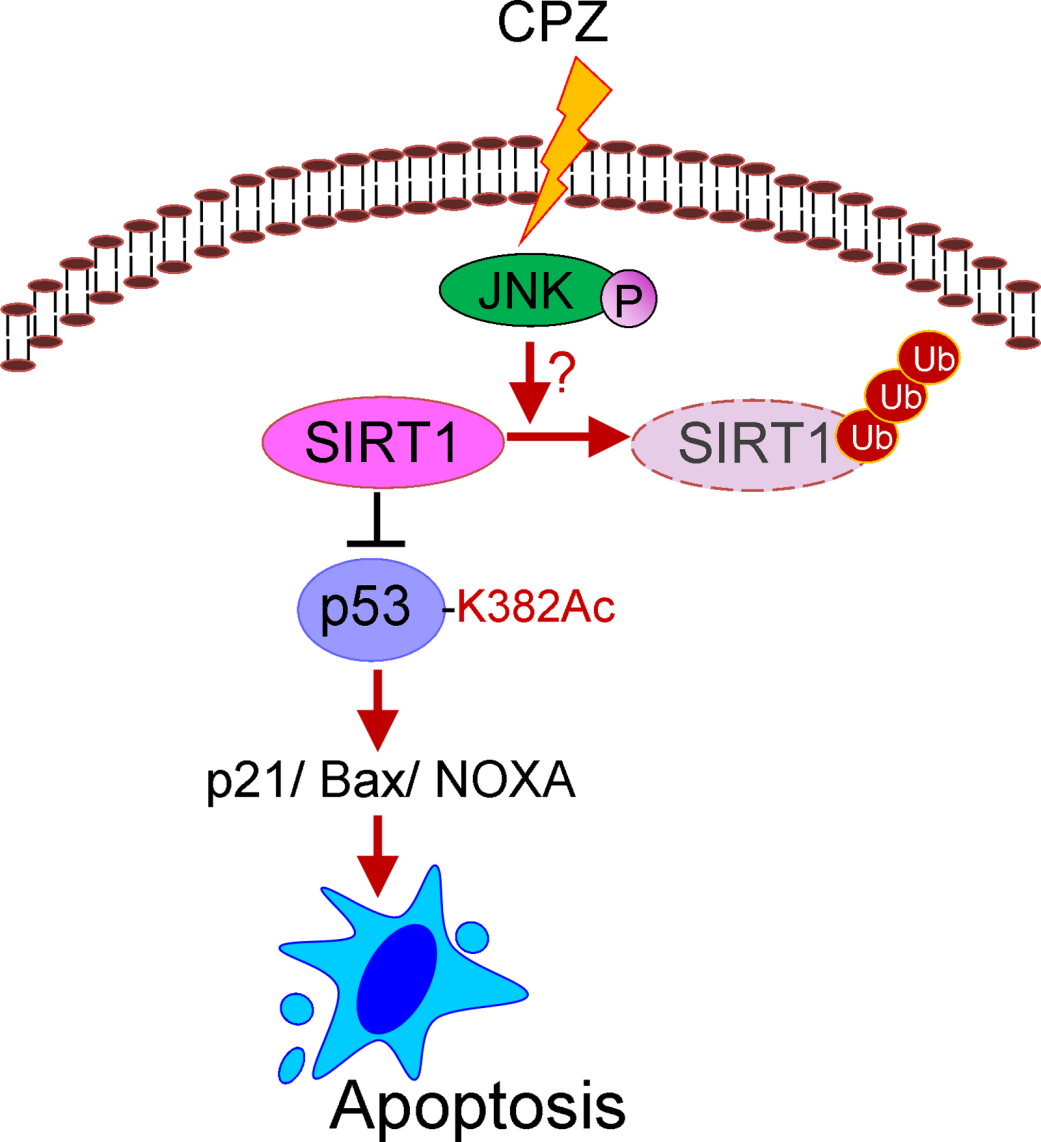
Schematic representation of the inhibitory mechanism of CPZ against CRC SIRT1 overexpression is associated with poor prognosis in CRC. SIRT1 inhibition by CPZ, through JNK-mediated SIRT1 protein degradation, facilitates p53 protein acetylation and downstream target expression, subsequently promoting tumor apoptosis.

Although the *TP53* tumor suppressor gene is frequently mutated in CRC, approximately 50% of cancer patients still have wild-type p53; thus, targeting the p53 pathway is a promising approach for cancer therapy. However, several p53 negative regulators, such as MDM2, MDMX, SIRT1, and HDACs, are abnormally elevated in tumor cells [8, 9], inactivating p53. Therefore, identifying drugs that can target these p53 suppressors and be used in monotherapy or combination therapy is an alternative approach [28–30]. However, traditional *de novo* drug discovery often takes more than 15 years, and the probability of success is lower than 10%. Repositioned drugs can be used to reduce the development time and risks because these drugs have already been used in clinics; thus, their toxicology, safety, and pharmacokinetic profiles have been studied extensively. In this study, we found that the antipsychotic drug CPZ effectively suppressed CRC growth by inducing the p53-dependent apoptotic process. CPZ induced JNK activation, which destabilized the SIRT1 protein level, increasing p53 acetylation and activation and consequently promoting tumor apoptosis (Figure 8).

CPZ is an FDA-approved antipsychotic drug used to treat schizophrenia and bipolar disorder and has shown potent antitumor activity in recent studies. CPZ inhibited cell-cycle progression in rat glioma C6 cells by inducing p21^Waf/Cip1^/Egr1 expression [25] and induced autophagic cell death by inhibiting the AKT/mTOR pathway in human glioma U87-MG cells [26]. In addition, CPZ enhanced therapeutic efficacy in tamoxifen-resistant breast cancer cells [31], suggesting that CPZ is a potential agent for cancer chemotherapy. We found that CPZ induced tumor apoptosis and suppressed xenograft tumor growth in colon cancer. Moreover, it induced tumor apoptosis in a p53-dependent manner. The inhibitory activity of CPZ showed specificity toward tumor cells and did not affect normal fibroblasts. Moreover, CPZ had low toxicity in mice and zebrafish embryos, suggesting that CPZ is highly toxic to tumor cells. Nevertheless, further studies are required to confirm these results.

SIRT1 is a NAD^+^-dependent deacetylase belongs to the class III HDAC. SIRT1 is upregulated in patients with prostate, breast, pancreatic, and colorectal cancers and plays a critical role in tumor initiation, progression, and drug resistance [32–35]. SIRT1 binds to and deacetylates p53, thereby downregulating p53 transcriptional activity. In addition to p53 deacetylation, Ku70, E2F1, and FOXO deacetylation by SIRT1 is also involved in blocking cellular senescence, differentiation, and apoptosis [36, 37]. Because of its overexpression in various malignancies and its role in preventing apoptosis, SIRT1 is attractive as a druggable target. For instance, tenovins and inauhzin inhibit SIRT1, thus increasing p53 activity and suppressing tumor growth [38, 39]. Inauhzin combined with chemotherapeutic agents sensitizes cells to p53-depedent cytotoxicity and tumor suppression [40]. Moreover, SIRT1 inhibition enhances the elimination of leukemia stem cells in combination with imatinib [41] and sensitizes natural compound-induced cell death in lung cancer cells [42]. SIRT1 was overexpressed in advanced-stage CRC tissues, and SIRT1 overexpression was associated with decreased survival in CRC patients, suggesting that high SIRT1 expression is associated with poor patient outcomes in CRC and that SIRT1 is an attractive therapeutic target for CRC. In the present study, CPZ-increased p53 Lys382 acetylation and transcriptional activity were associated with SIRT1 inhibition, whereas ectopic SIRT1 expression abolished CPZ-mediated p53 activation and tumor apoptosis. Acetylation of p53 prevents its degradation by MDM2 and increases its transcriptional activity [4]. In addition, acetylated p53 is required for Bax activation through transcription-independent apoptosis regulation [43]. Furthermore, CPZ reduced the SIRT1 protein level in both p53 wild-type (HCT116 and LoVo) and mutant (HCT15) cells (Supplementary Figure S2B), suggesting that CPZ is a SIRT1 inhibitor and can treat p53 mutant CRC cells by attenuating other SIRT1-dependent oncogenic signaling pathways; however, additional evidence supporting these inferences is required.

SIRT1 activity can be regulated through posttranslational modifications such as phosphorylation and sumoylation [44, 45]. Previous studies have reported that AMP-activated protein kinase phosphorylates and inactivates SIRT1, resulting in increased p53 acetylation in liver cancer [46, 47]. Similarly, JNK activation induced by insulin treatment promotes SIRT1 degradation and inactivation in hepatocytes [48]. By contrast, Nasrin and colleagues reported that JNK phosphorylates and increases SIRT1 enzymatic activity in response to oxidative stress [49], contradicting our finding that JNK activation is crucial for CPZ-mediated SIRT1 ubiquitination and degradation. Our data showed that treatment with a JNK inhibitor suppressed CPZ-induced tumor apoptosis and abolished CPZ-elicited SIRT1 degradation and p53 acetylation. In addition, JNK activation has been demonstrated to be crucial for p53 protein phosphorylation and transcriptional activity. Our study showed that CPZ activated JNK, at least partly, by inhibiting the SIRT1 protein level, thus increasing p53 acetylation and promoting tumor apoptosis. In conclusion, our study provides new insight into the repositioning of antipsychotic drugs for CRC treatment. CPZ exerts proapoptotic activity in CRC through JNK-mediated SIRT1 inhibition and p53 activation and could be a promising therapeutic agent and supplement for CRC treatment. Moreover, the potential interactions between CPZ and other conventional anti-CRC drugs are worth investigating.

## MATERIALS AND METHODS

### Chemicals and cell lines

CPZ was purchased from Sigma Chemical Co. PD98059, SB203580, SP600125, and MG132 were obtained from Merck Millipore. The human CRC cell lines HCT116 (wild-type p53), LoVo (wild-type p53), HCT15 (mutant p53), and HT29 (mutant p53) were purchased from the Bioresource Collection and Research Center (Hsinchu, Taiwan). The HCT116 cells were maintained in Dulbecco's modified Eagle's medium. The HCT15, HT29, and LoVo cells were maintained in RPMI at 37°C in a humidified incubator containing 5% CO_2_. All culture media were supplemented with 7% heat-inactivated FBS, 100 units (U)/mL penicillin, and 100 U/mL streptomycin (Gibco). The cell lines were authenticated using a short tandem repeat profiling analysis to ensure that no culture contamination occurred.

### Plasmids and transfection

Wild-type and mutant (H363Y) SIRT1-expressing vectors (gifts from Michael Greenberg; Addgene #1791 and #1792) were subcloned into the pCMV6-entry vector (Origene). To establish a stable SIRT1-knockdown cell line, HEK293T cells were cotransfected with hairpin pLKO-shSIRT1 vectors (National RNAi Core Facility, Academia Sinica, Taipei, Taiwan), a packaging plasmid (pCMV-ΔR8.91), and an envelope (pMDG) plasmid. Transduced cells were selected using puromycin (2 μg/mL) [50]. To measure the effect of CPZ on p53 activity, HCT116 cells were seeded into 24-well plates and transfected with a PG13-luc plasmid containing 13 copies of the wtp53-binding sites (a gift from Bert Vogelstein; Addgene plasmid #16442). CPZ was then added, the cells were incubated for 18 h, and p53 activity was measured using a luciferase assay kit (Promega).

### Cell viability

Cells were seeded into 24-well plates at a concentration of 1 × 10^5^ cells per well. The cells were refed with a complete growth medium containing CPZ (0–40 μM) or pretreated for 1 h with caspase 3 inhibitors (z-DEVD-FMK) or MAPK (PD98059, SP600125, and SB203580). After 24–48 h of incubation, the growth medium was removed and the cells were refed with a growth medium containing MTT (50 μg/mL) and incubated further for 1 h. Formazan crystals were dissolved in isopropanol, and absorbance was determined using an ELISA reader (Dynatech MR-7000; Dynatech Laboratories) at a wavelength of 600 nm. Cell viability was expressed as a percentage of untreated control cells.

### Apoptosis detection

HCT116 cells (10^5^/well) were seeded onto cover slides and treated with CPZ for 24 h. The slides were fixed in methanol and stained with DAPI at room temperature for 30 min. Apoptotic nuclei were observed using a fluorescent microscope and determined by calculating the percentage of bright and condensed nuclei among the total number of cells. To detect cellular apoptosis, the HCT116 cells were treated with CPZ for 24 h and incubated with annexin V-fluorescein isothiocyanate and PI solutions (BD Biosciences) according to the manufacturer's instructions. The cells were analyzed using the FACSCanto II flow cytometry system and FACSDiva software v6.1 (BD Biosciences).

### Western blotting

The cells were lysed using a radioimmunoprecipitation assay buffer (150 mM NaCl, 50 mM Tris-HCl at pH 7.4, 1% Nonidet P-40, 0.5% sodium deoxycholate, and 0.1% SDS) containing a protease and phosphatase inhibitor cocktail (Roche). Equal amounts of proteins were separated using SDS-PAGE and then transferred onto polyvinylidene difluoride membranes (Millipore). The membranes were blocked with 3% bovine serum albumin/TBST and incubated overnight with specific primary antibodies. The membranes were then incubated with appropriate horseradish peroxidase (HRP)-conjugated secondary antibodies (Genetex) for 1 h at room temperature, and proteins were detected using an enhanced chemiluminescence kit (Millipore). Protein expression was quantified using ImageJ software. Data are expressed as the mean ± standard error (SE) and represented as fold changes relative to the control, *n* = 3. Details on the antibodies used are listed in Supplementary Table S1.

### Real-time PCR

Total RNA was extracted using an RNeasy Plus Mini Kit (Qiagen) and reverse transcribed using SuperScript III reverse transcriptase (Invitrogen). Quantitative PCR was performed using the resulting cDNA, a LightCycler 480 SYBR Green I Master Mix (Roche), and a LightCycler 480 System (Roche). The results were calculated using the equation for ΔΔC_T_ and are expressed as fold changes relative to the control sample. *GAPDH* was used as an internal control for normalization. Specific primer sequences are listed in Supplementary Table S2.

### Animal study

HCT116 (2 × 10^6^) cells were injected into the dorsal flank of 6- to 8-week-old NOD/SCID mice (*n* = 6 in each group). When the tumor volume reached 300 mm^3^, the mice were intravenously administered CPZ (10 mg/kg) or PBS (vehicle control) every 2 or 3 days for 3 weeks. The body weights and tumor burdens of the mice were monitored every 3 days during the experiments. Tumor volume was calculated using the following equation: length × (width)^2^ × 0.52. All animals were cared for in a specific-pathogen-free room and treated in accordance with the animal care protocol approved by an animal committee.

### Immunohistochemistry

A colon cancer tissue microarray (BC51110) was purchased from Biomax. The slides were deparaffinized, rehydrated, and heated in a sodium citrate buffer (0.01 M, pH 6.0) for antigen retrieval. The slides were incubated with 3% hydrogen peroxide in methanol and 1% BSA to block endogenous peroxidase activity and nonspecific binding, respectively. The slides were then incubated overnight with SIRT1 antibodies at 4°C and washed three times by using PBS with 0.1% Tween 20. The slides were treated with a polymer-HRP IHC detection reagent (BioGenex), and 3, 3′-diaminobenzidine was used as a chromogen to visualize peroxidase activity.

### Clinical relevance of SIRT1

Two CRC microarray data sets, GSE14333 [51] and GSE17536 [52], were downloaded from the Gene Expression Omnibus website (http://www.ncbi.nlm.nih.gov/geo), and data on the SIRT1 mRNA level (probe ID: 218878_s_at) were extracted. SIRT1 mRNA levels higher than the average value were classified into a SIRT1-high group, whereas others were classified into a SIRT1-low group. A Kaplan–Meier survival curve was constructed using GraphPad Prism 5 software. *P* values were analyzed using the log-rank (Mantel–Cox) statistical test.

### Statistical analyses

All data presented in the current study are representative of three independent experiments, and the values are expressed as the mean ± SE. The protein bands were quantified using densitometry (ImageJ software). The significance of the difference from the respective controls for each test condition in each paired experiment was assessed using the Student *t* test. A *P* value of < 0.01 or < 0.05 was considered statistically significant.

## SUPPLEMENTARY FIGURES AND TABLES


