# Supplementary material for: Repositioning antipsychotic chlorpromazine for treating colorectal cancer by inhibiting sirtuin 1
Source: Oncotarget. 2015 Sep 5;6(29):27580–95. doi: 10.18632/oncotarget.4768 (PMC4695010; doi:10.18632/oncotarget.4768)
Supplement: Supplementary file 1 [file oncotarget-06-27580-s001.pdf]

SUPPLEMENTARY FIGURES AND TABLES

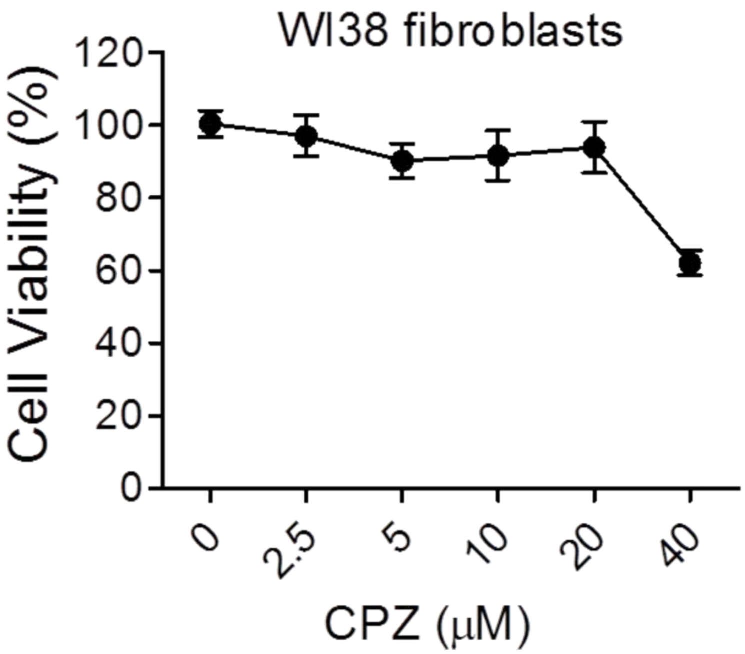

Supplementary Figure S1: Cytotoxic effect of CPZ on normal fibroblast. Human lung embryonic fibroblast WI38 cells were treated with CPZ (0–40 μM) for 24 h, and cell viability was determined by MTT assay.

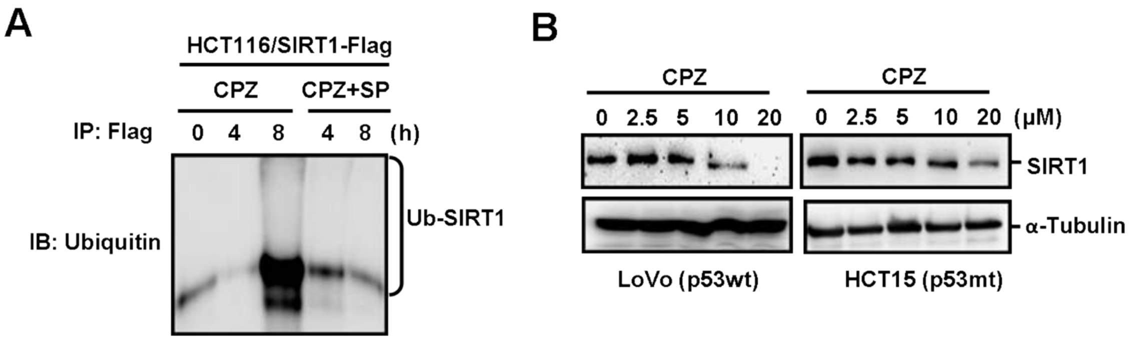

Supplementary Figure S2: Effect of CPZ on SIRT1 protein level in colorectal cancer cells. A. HCT116/SIRT1-Flag cells were treated with CPZ alone, CPZ+SP600125 for 0-8 h, and protein lysates were precipitated with anti-Flag antibody and then blotted with anti-ubiquitin antibody. **B.** HCT15 and LoVo cells were treated with CPZ (0–40 μM) for 24 h, and SIRT1 protein level was analyzed by Western bot.

Supplementary Table S1. List of antibodies and its characteristic

Antigen	Antibody	Experimental conditions
Flag (DDDDK)	GeneTex (GTX115043)	WB (1:5000) IP (1:500)
P53	Santa Cruz (sc126)	WB (1:5000)
P21	GeneTex (GTX112898)	WB (1:3000)
SIRT1	Santa Cruz (sc15404)	WB (1:3000)
SIRT1	Abcam (ab32441)	IHC (1:150)
P53K382Ac	Cell Signaling (#2525)	WB (1:3000)
P53K382Ac	Abcam (GTX62061)	IHC (1:100)
Caspase3	GeneTex (GTX110543)	WB (1:3000)
PARP	GeneTex (GTX100573)	WB (1:3000)
BAX	GeneTex (GTX109683)	WB (1:3000)
Mcl-1	GeneTex (GTX102026)	WB (1:3000)
Bcl-x	GeneTex (GTX100632)	WB (1:3000)
p-JNK	Cell Signaling (#9251)	WB (1:2000)
t-JNK	Cell Signaling (#9252)	WB (1:5000)
α-Tubulin	GeneTex (GTX113617)	WB (1:10000)
Ubiquitin	Cell Signaling (#3936)	WB (1:2000)
PCNA	GeneTex (GTX100539)	WB (1:3000)

Abbreviations: IHC, immunohistochemistry; IP, immunoprecipitation; WB, Western blot.

Supplementary Table S2. List of oligonucleotides for real-time PCR

Assay	Gene	Sequence (5′ → 3′)
Real-time PCR	TP53	F: TAACAGTTCCTGCATGGGCGGCR: AGGACAGGCACAAACACGCACC
	P21	F: AAGACCATGTGGACCTGTCACTGTR: AGGGCTTCCTCTTGGAGAAGATCA
	NOXA	F: ACCAAGCCGGATTTGCGATTR: ACTTGCACTTGTTCCTCGTGG
	BAX	F: CCAAGAAGCTGAGCGAGTGR: GTCCACGGCGGCAATCATC
RT-PCR	SIRT1	F: TCCTGGACAATTCCAGCCATCTCTR: TTCCAGCGTGTCTATGTTCTGGGT
	GAPDH	F: AGCCACATCGCTCAGACACR: GCCCAATACGACCAAATCC
